# Supplementary material for: Development of a Prediction Model for Severe Hypoglycemia in Children and Adolescents with Type 1 Diabetes: The Epi-GLUREDIA Study
Source: Nutrients. 2025 Aug 12;17(16):2610. doi: 10.3390/nu17162610 (PMC12389539; doi:10.3390/nu17162610)
Supplement: Supplementary file 1 [file nutrients-17-02610-s001.zip › nutrients-3766303-supplementary.pdf]

Table S1. Results of univariate analyses.

Variable 1	Variable 2	Statistical test	Statistic	P-value
Hypo. frequency	$TBR_{<70}$	Spearman	0,92	<0.001***
$TAR_{>180}$	TIR_{70-180}	Spearman	-0,93	<0.001***
$TAR_{>180}$	Glycemic mean	Spearman	0,98	<0.001***
$TAR_{>180}$	PC1	Spearman	-0,97	<0.001***
TIR_{70-180}	Glycemic mean	Spearman	-0,89	<0.001***
TIR_{70-180}	PC1	Spearman	0,89	<0.001***
Glycemic mean	PC1	Spearman	-1,00	<0.001***
$TBR_{<70}$	CV	Spearman	0,68	<0.001***
Hypo. frequency	CV	Spearman	0,64	<0.001***
Age	Age at diagnosis	Spearman	0,41	<0.001***
Hypo. frequency	PC1	Spearman	0,41	<0.001***
Hypo. frequency	Glycemic mean	Spearman	-0,40	<0.001***
Hypo. frequency	$TAR_{>180}$	Spearman	-0,37	<0.001***
Gender	Age at diagnosis	Kruskal	211,99	<0.001***
$TBR_{<70}$	PC1	Spearman	0,34	<0.001***
$TBR_{<70}$	Glycemic mean	Spearman	-0,33	<0.001***
$TBR_{<70}$	$TAR_{>180}$	Spearman	-0,30	<0.001***
CV	Age	Spearman	0,26	<0.001***
TIR_{70-180}	CV	Spearman	-0,25	<0.001***
Last glycemic data	$TBR_{<70}$	Kruskal	47,76	<0.001***
TIR_{70-180}	Age	Spearman	-0,18	<0.001***
$TBR_{<70}$	Age at diagnosis	Spearman	0,17	<0.001***
Last glycemic data	Hypo. frequency	Kruskal	36,13	<0.001***
Last glycemic data	CV	Kruskal	30,82	<0.001***
CV	Age at diagnosis	Spearman	0,14	<0.001***
Gender	Age	Kruskal	26,00	<0.001***
Glycemic mean	Age	Spearman	0,13	<0.001***
Age	PC1	Spearman	-0,13	<0.001***
$TBR_{<70}$	Age	Spearman	0,13	<0.001***
Hypo. frequency	TIR_{70-180}	Spearman	0,12	<0.001***
Hypo. frequency	Age at diagnosis	Spearman	0,12	<0.001***
$TAR_{>180}$	Age	Spearman	0,12	<0.001***
Hypo. frequency	Age	Spearman	0,11	<0.001***
CV	PC5	Spearman	-0,09	<0.01**
Age	PC5	Spearman	-0,07	<0.05*
Glycemic mean	CV	Spearman	0,07	<0.05*
Age	PC3	Spearman	-0,07	<0.05*
Last glycemic data	PC4	Kruskal	6,46	<0.05*
CV	PC1	Spearman	-0,07	<0.05*
$TBR_{<70}$	Gender	Kruskal	6,33	<0.05*
Last glycemic data	$TAR_{>180}$	Kruskal	6,32	<0.05*
Last glycemic data	Glycemic mean	Kruskal	6,01	<0.05*
Last glycemic data	PC1	Kruskal	5,90	<0.05*

$TBR_{<70}$	PC2	Spearman	-0,06	<0.05*
Last glycemic data	PC5	Kruskal	5,02	0,06
Hypo. frequency	PC3	Spearman	-0,06	0,07
CV	PC2	Spearman	-0,05	0,08
Glycemic mean	Age at diagnosis	Spearman	-0,05	0,11
Hypo. frequency	PC2	Spearman	-0,05	0,11
Age at diagnosis	PC1	Spearman	0,05	0,11
$TAR_{>180}$	Age at diagnosis	Spearman	-0,05	0,13
Last glycemic data	PC2	Kruskal	3,35	0,13
$TBR_{<70}$	PC3	Spearman	-0,05	0,14
Hypo. frequency	Gender	Kruskal	3,11	0,15
Age	PC2	Spearman	-0,05	0,16
$TAR_{>180}$	Gender	Kruskal	2,96	0,16
Glycemic mean	Gender	Kruskal	2,77	0,18
Gender	PC1	Kruskal	2,68	0,19
Age	PC4	Spearman	0,04	0,19
CV	PC3	Spearman	-0,04	0,19
Gender	PC3	Kruskal	2,57	0,20
Last glycemic data	Age at diagnosis	Kruskal	2,41	0,21
Gender	PC2	Kruskal	2,12	0,25
Hypo. frequency	PC4	Spearman	-0,03	0,33
TIR_{70-180}	Age at diagnosis	Spearman	-0,03	0,36
Hypo. frequency	PC5	Spearman	-0,03	0,37
$TAR_{>180}$	CV	Spearman	0,03	0,38
PC2	PC3	Spearman	0,03	0,39
Age at diagnosis	PC4	Spearman	0,03	0,41
Age at diagnosis	PC2	Spearman	0,03	0,44
Age at diagnosis	PC3	Spearman	0,03	0,45
Last glycemic data	PC3	Kruskal	0,82	0,54
TIR_{70-180}	Gender	Kruskal	0,69	0,59
TIR_{70-180}	PC3	Spearman	0,02	0,64
TIR_{70-180}	PC2	Spearman	0,02	0,68
Gender	PC5	Kruskal	0,45	0,68
$TBR_{<70}$	PC4	Spearman	-0,02	0,69
$TBR_{<70}$	TIR_{70-180}	Spearman	0,02	0,71
$TBR_{<70}$	PC5	Spearman	-0,02	0,73
$TAR_{>180}$	PC3	Spearman	-0,01	0,76
$TAR_{>180}$	PC5	Spearman	0,01	0,79
Glycemic mean	PC3	Spearman	-0,01	0,81
Glycemic mean	PC2	Spearman	-0,01	0,83
PC2	PC5	Spearman	0,01	0,84
PC3	PC4	Spearman	-0,01	0,88
Last glycemic data	Gender	Fisher	0,86	0,88
$TAR_{>180}$	PC2	Spearman	-0,01	0,88
Last glycemic data	TIR_{70-180}	Kruskal	0,10	0,88
Gender	PC4	Kruskal	0,09	0,88
PC1	PC4	Spearman	-0,01	0,88

PC1	PC5	Spearman	-0,01	0,88
Age at diagnosis	PC5	Spearman	0,01	0,89
Glycemic mean	PC4	Spearman	0,01	0,92
TIR_{70-180}	PC5	Spearman	-0,01	0,92
PC1	PC2	Spearman	0,01	0,92
$TAR_{>180}$	PC4	Spearman	0,00	0,97
CV	PC4	Spearman	0,00	0,98
TIR_{70-180}	PC4	Spearman	0,00	0,98
PC1	PC3	Spearman	0,00	0,98
PC3	PC5	Spearman	0,00	0,98
CV	Gender	Kruskal	0,00	0,98
Last glycemic data	Age	Kruskal	0,00	0,98
Glycemic mean	PC5	Spearman	0,00	0,98

Results of univariate analyses. Variable 1 and Variable 2 refer to the variables under investigation. Statistical test indicates the type of test performed, and Statistic reports the corresponding test value. Statistical significance was set at $p < 0.05$. The significance levels are represented as follows: not significant (), $p < 0.05$ (*), $p < 0.01$ (**), $p < 0.001$ (***). The variables are defined as follows: Age, patient's age at the time of the study (years); Age at diagnosis, patient's age at diagnosis (years); CV, coefficient of glycemic variability (%); Gender, patient's gender; Glycemic mean, mean glucose value in the dataset (mg/dL); Hypo. Frequency, frequency of hypoglycemic events in the dataset (n/day); PC1–PC5, principal components 1 to 5 from the PCA; $TAR_{>180}$, percentage of time spent in hyperglycemia (> 180 mg/dL); $TBR_{<70}$, percentage of time spent in hypoglycemia (< 70 mg/dL); $TIR_{70–180}$, percentage of time spent in target glucose range (70–180 mg/dL).
